# High thermal conductivity in metallic *θ*-TaN single crystals

**DOI:** 10.1093/nsr/nwag106

**Published:** 2026-02-14

**Authors:** Yizhe Liu, Xuefeng Zhou, Guijian Pang, Chao Gu, Guozhu Song, Jian Chen, Jesús Carrete, Leiming Fang, Shanmin Wang, Wu Li, Bo Sun

**Affiliations:** Tsinghua SIGS, Tsinghua University, Shenzhen 518055, China; Department of Physics, Guangdong Basic Research Center of Excellence for Quantum Science, and State Key Laboratory of Quantum Functional Materials, Southern University of Science and Technology, Shenzhen 518055, China; Center for High Pressure Science & Technology Advanced Research, Beijing 100094, China; Eastern Institute for Advanced Study, Eastern Institute of Technology, Ningbo 315200, China; Department of Applied Physics, The Hong Kong Polytechnic University, Hong Kong, China; Department of Physics, Guangdong Basic Research Center of Excellence for Quantum Science, and State Key Laboratory of Quantum Functional Materials, Southern University of Science and Technology, Shenzhen 518055, China; Department of Physics, Guangdong Basic Research Center of Excellence for Quantum Science, and State Key Laboratory of Quantum Functional Materials, Southern University of Science and Technology, Shenzhen 518055, China; Department of Physics, Guangdong Basic Research Center of Excellence for Quantum Science, and State Key Laboratory of Quantum Functional Materials, Southern University of Science and Technology, Shenzhen 518055, China; Instituto de Nanociencia y Materiales de Aragón, CSIC-Universidad de Zaragoza, Zaragoza E-50009, Spain; National Key Laboratory of Neutron Science and Technology, Institute of Nuclear Physics and Chemistry, China Academy of Engineering Physics, Mianyang 621900, China; Department of Physics, Guangdong Basic Research Center of Excellence for Quantum Science, and State Key Laboratory of Quantum Functional Materials, Southern University of Science and Technology, Shenzhen 518055, China; Eastern Institute for Advanced Study, Eastern Institute of Technology, Ningbo 315200, China; Institute for Advanced Study, Shenzhen University, Shenzhen 518060, China; Tsinghua SIGS, Tsinghua University, Shenzhen 518055, China; Guangdong Provincial Key Laboratory of Thermal Management Engineering & Materials, Shenzhen 518055, China

**Keywords:** high thermal conductivity, metallic materials, phonon–electron coupling

## Abstract

Metallic materials are critical in integrated circuits as they not only deliver electricity but also dissipate heat. However, their performance is constrained as the thermal conductivity of metals is capped with a value of ∼400 W m^−^^1^ K^−^^1^. Here, we shatter this long-standing ceiling by high-pressure synthesis of the metallic hexagonal tantalum mononitride (*θ*-TaN) single crystal with ultrahigh thermal conductivity. The synthesized *θ*-TaN single crystal exhibits a room-temperature thermal conductivity of 502 W m^−^^1^ K^−^^1^, exceeding the conventional upper limit for metallic thermal conductors, despite the presence of a substantial concentration of nitrogen vacancies. Our findings identify a clear pathway for further enhancing thermal conductivity through minimizing vacancy concentration in *θ*-TaN. This work establishes *θ*-TaN as a highly promising candidate for advanced thermal management applications and introduces a new approach for designing metallic conductors to surpass conventional limits.

## Teaser

We synthesized a new metallic material, *θ*-TaN with ultrahigh thermal conductivity, surpassing all conventional metals, which opens new opportunities for next-generation thermal management strategies.

## INTRODUCTION

In the architecture of semiconductor devices, metallic materials play a critical, multifaceted role [1,2]. Beyond their mission as electrical interconnects to deliver power and signals, they also serve as critical conduits to dissipate damaging waste heat [2,3]. In metals, the lattice contribution to thermal conductivity is usually negligible, and the electronic thermal conductivity is proportional to the electrical conductivity, as formalized by the Wiedemann–Franz law. Thus, silver and copper are metals with the highest thermal conductivity of ∼400 W m^−^^1^ K^−^^1^ [4,5]. This performance is dwarfed by non-metallic counterparts like diamond [6] and boron arsenide (BAs) [7–9], which exhibit thermal conductivities many times higher. In metallic materials, the intrinsically limited lattice thermal conductivity originates mainly from the strong lattice anharmonicity scattering and phonon–electron interactions [10]. While the former predominantly governs the phonon scattering process at high temperatures, the latter mechanism becomes dominant at low temperatures or in systems exhibiting inherently weak phonon–phonon coupling, such as in tungsten [11] and some transition-metal (TM) carbides [12,13]. While metallic graphite is an exception with a high in-plane thermal conductivity of ∼2000 W m^−^^1^ K^−^^1^, the low cross-plane value and poor processability limit its practical applications [14]. Therefore, the pursuit of metallic materials with high thermal conductivity is not merely a search for a scientific breakthrough but also represents the critical path to shattering the thermal bottleneck and enabling the future of high-power, high-density electronics.

Metallic transition-metal nitrides and carbides have been considered prime candidates to overcome the aforementioned limitations [13,15] due to their unique electronic and phononic structures. The large mass ratio of constituent atoms provides a wide frequency gap between acoustic and optical phonons, along with acoustic phonon bunching, which effectively suppresses the

intrinsic phonon–phonon scattering rate. Meanwhile, the low electronic density of states near the Fermi level yields a correspondingly small carrier population, which inherently suppresses the phonon–electron scattering rate. Importantly, some of us have previously predicted that metallic *θ*-TaN has an extraordinary thermal conductivity as high as 995 W m^−^^1^ K^−^^1^ [15]. Despite the promising theoretical predictions, the synthesis of high-quality *θ*-TaN single crystals has remained a longstanding challenge. This difficulty originates from the harsh synthesis conditions of high pressures and high temperatures identified by the established phase diagram [16]. Specifically, high growth temperatures typically induce nitrogen degassing in TM nitrides [17]. Furthermore, kinetic constraints in the solid state would restrict the atomic diffusion rate, leading to incomplete chemical reactions [18]. These factors critically impede the production of high-quality, near-stoichiometric TM nitride single crystals. Consequent structural imperfections would strongly scatter heat-carrying phonons or electrons, thus limiting the achievement of its intrinsic ultrahigh thermal conductivity.

In this work, we successfully synthesized metallic *θ*-TaN single crystals using a high-pressure, high-temperature method. We measured a room-temperature thermal conductivity of 502 W m^−^^1^ K^−^^1^, a value that surpasses all conventional metals, which establishes *θ*-TaN as the new ceiling for metallic thermal conductors. The temperature-dependent thermal conductivity deviates from *T*^−^^1^, suggesting the existence of atomic deficiencies that introduce additional phonon -defect scattering to suppress thermal conductivity, which is confirmed by our neutron diffraction measurement. This finding demonstrates the untapped potential of *θ*-TaN and provides a new strategic direction for future heat dissipation technologies, where engineering defect-free crystalline structures will be paramount to unlocking thermal transport properties.

## RESULTS AND DISCUSSION

**Figure 1.**
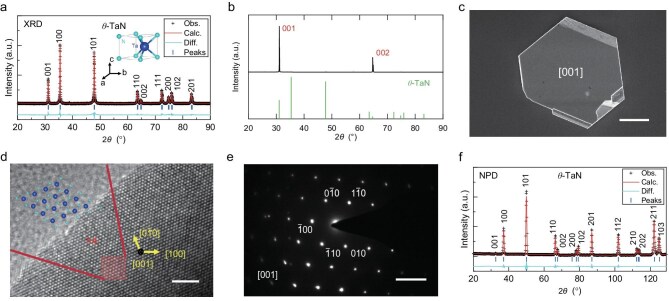
Structure of the synthesized *θ*-TaN. (a) Refined powder XRD pattern. (b) XRD pattern of a number of single crystals aligned along the [001] direction. (c) SEM image. (d) HRTEM image. (e) SAED patterns. (f) Refined NPD patterns. Scale bars: 10 μm (c), 2 nm (d), and 5 1/nm (e).

We synthesize *θ*-TaN single crystals through a high-pressure, high-temperature nitridation reaction between sodium amide (NaNH_2_) and Ta powder, as detailed in Supplementary Data [19]. Initial characterization of the product by powder X-ray diffraction (XRD) confirmed a phase-pure material, and all the diffraction peaks can be indexed by the hexagonal *P*${\mathrm{\bar{6}}}$*m*2 structure (Fig. 1a), which is structurally isotypic with tungsten carbide (WC). The refined lattice parameters are *a =* 2.9337 Å and *c =* 2.8832 Å, which is in agreement with previous reports [15,20]. Our synthesis yields high-quality *θ*-TaN single crystals oriented along the c-axis. The single-crystalline nature is unequivocally established by single-crystal XRD, where patterns are dominated by a series of sharp (00*l*) reflections, confirming a *c*-axis orientation normal to the crystal’s basal plane (Fig. 1b). This hexagonal symmetry manifests in a characteristic plate-like morphology, with crystals typically reaching ∼30 μm in lateral size and ∼3 μm in thickness (Fig. 1c), similar to other hexagonal TM nitrides [21,22]. At the atomic level, high-resolution transmission electron microscopy (HRTEM) reveals a perfectly ordered lattice consistent with the *P*${\mathrm{\bar{6}}}$*m*2 symmetry (Fig. 1d). This single-crystalline structure is further corroborated by the corresponding selected-area electron diffraction (SAED) pattern, which displays a pristine hexagonal array of diffraction spots indexed along the [001] zone axis (Fig. 1e). Together, these comprehensive characterizations demonstrate excellent crystallinity and phase purity of the as-synthesized *θ*-TaN crystals.

However, due to the insensitivity of X-rays to light elements (like nitrogen), the overall XRD signals of TaN are predominantly dictated by the tantalum sublattice, which does not allow an accurate determination of atomic positions and deficiencies. In this regard, neutron diffraction is a powerful tool to probe nitrogen atoms, because the coherent scattering length of the nitrogen atom is as large as 9.36 fm, which is ∼36% larger than that of the tantalum atom (i.e., 6.91 fm), meaning that the nitrogen atom is much more sensitive to neutron scattering than that of the tantalum atom. Thus, the crystal structures can be more readily refined by neutron diffraction. We therefore conducted neutron powder diffraction (NPD) measurements, as shown in Fig. 1f. The significant differences in reflection intensities between the NPD and XRD patterns, especially for the 001 and 111 peaks, are a direct consequence of different sensitivities of tantalum and nitrogen atoms to X-ray and neutron probes, as observed for many TM nitrides [21,23,24]. The optimum refinement can be reached with the involvement of disordered nitrogen vacancies, with an average concentration of 1%. Obviously, the presence of nitrogen vacancies does not alter the overall symmetry of the crystal as the involved vacancies are disordered, in analogy with that of molybdenum nitrides and tungsten nitrides with the cF8 phase [21,22]. The detailed crystal structure of the synthesized *θ*-TaN is summarized in Table S1.

**Figure 2.**
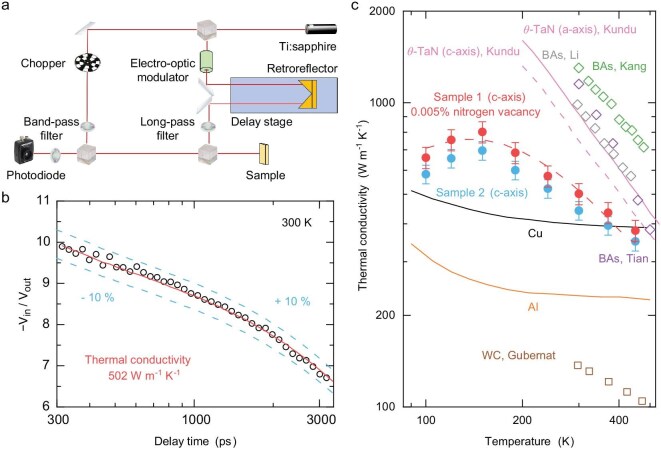
Thermal conductivity measurements on the synthesized *θ*-TaN. (a) Schematic of the TDTR setup. (b) Representative TDTR data (open black circles) at 300 K. Experimental data were fitted using a multilayer thermal model. The fitting uncertainty is illustrated by plotting the thermal conductivity with a ±10% variation (dashed blue lines) from the best-fit curve (solid red line). (c) Measured temperature-dependent thermal conductivity along the *c*-axis (solid red circles for Sample 1 and solid blue circles for Sample 2), compared to experimental values of WC (open brown squares) [60], Al (solid orange line) [4], Cu (solid black line) [4], and BAs reported by Kang *et al*. [9] (open green diamonds), Li *et al*. [8] (open gray diamonds), and Tian *et al*. [7] (open purple diamonds). The dashed red line denotes the lattice thermal conductivity along the *c*-axis from our first-principles calculations, considering the combination of three-phonon scattering, four-phonon scattering, phonon -electron scattering, phonon -isotope scattering, phonon–boundary scattering (7 μm), and phonon -defect scattering (0.005% nitrogen vacancy). Reported calculations of thermal conductivity in pure *θ*-TaN along the *a* and *c* axes are shown as a solid pink line and a dashed pink line, respectively [15].

We then measured the thermal conductivity of the synthesized *θ*-TaN single crystals using time-domain thermoreflectance (TDTR) [25,26], a well-established pump-probe technique to extract a materials’ thermal properties [27–33]. Our TDTR setup is schematically illustrated in Fig. 2a, and detailed information on our TDTR measurements is provided in Supplementary Data. We show the typical TDTR data acquired at 300 K in Fig. 2b, where we measured an ultrahigh thermal conductivity of 502 W m^−^^1^ K^−^^1^ for the synthesized *θ*-TaN single crystal with an experimental uncertainty <±10%. The measured thermal conductivity is significantly higher than previously reported results for polycrystalline *θ*-TaN samples [23,34]. More importantly, this value is remarkably higher than that of all conventional metals [4,5], positioning *θ*-TaN as the best metallic thermal conductor without significant anisotropy. While the as-synthesized crystals exhibited some variation in quality, all samples measured displayed thermal conductivities (from 442 to 502 W m^−^^1^ K^−^^1^) higher than that of pure copper (Fig. S4), challenging the conventional perception that the thermal conductivity of metals is inherently restricted and capped.

To explore the origin of the ultrahigh thermal conductivity, we conducted first-principles calculations on *θ*-TaN, incorporating three-phonon scattering, four-phonon scattering, phonon -isotope scattering, and phonon–electron scattering (see Supplementary Data for details). Our calculations indicated that phonon -electron scattering in *θ*-TaN is significantly weaker compared to conventional metals and semimetallic materials. Furthermore, phonon bunching in the acoustic region results in a considerably large energy gap between acoustic and optical phonons, thus limiting the three-phonon scattering, especially at intermediate phonon energies. The combination of these two mechanisms is responsible for the ultrahigh thermal conductivity in our *θ*-TaN. Despite the measured ultrahigh value, we notice that the thermal conductivity of our *θ*-TaN crystals is only ∼60% of our calculated value along the *c*-axis, considering our measurements are most sensitive to *c*-axis thermal conductivity (Fig. S2). This result suggests that additional mechanisms exist to effectively scatter phonons. Based on the presence of nitrogen vacancies revealed by NPD refinement, we attribute the reduced thermal conductivity to strong phonon -defect scattering by nitrogen vacancies, which effectively decreases the phonon mean free path and subsequently suppresses thermal conductivity.

**Figure 3.**
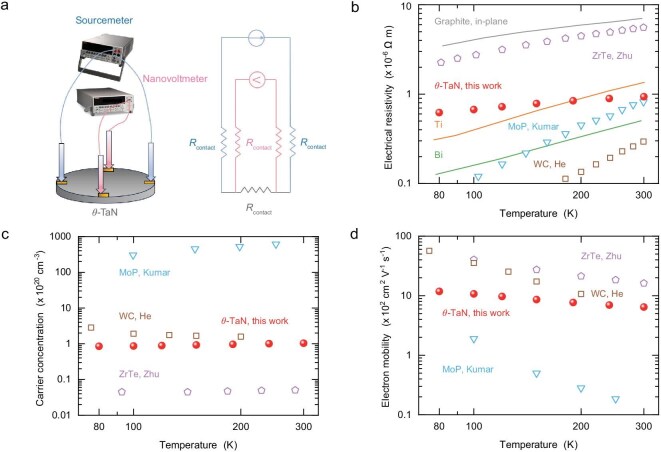
Electrical measurements on the synthesized *θ*-TaN (solid red circles), compared to experimental values of MoP [40] (open blue triangles), WC [39] (open brown squares), and ZrTe [41] (open purple pentagons). (a) Schematic of the electrical measurement setup. (b) Electrical resistivity, comparing the measured values in titanium (orange line) [44], bismuth (green line) [43], and graphite along the in-plane direction (gray line) [45]. (c) Carrier concentration. (d) Electron mobility.

The influence of these vacancies was investigated experimentally through temperature-dependent TDTR measurements (Fig. 2c). Considering the variability in crystal quality, we performed measurements on two representative crystals, namely Sample 1 and Sample 2, which correspond to the crystals displayed in Fig. S4a and S4d, respectively. Both samples exhibit a consistent temperature dependence of the thermal conductivity, although the absolute values show a small divergence due to differing sample quality. Consequently, the following analysis and discussion will focus primarily on the data set obtained from Sample 1. The maximum thermal conductivity of 812 W m^−^^1^ K^−^^1^ is measured at 150 K. At higher temperatures, the thermal conductivity decreases with temperature, following a *T*^−^^0.7^ dependence, which deviates from the typical *T*^−^^1^ trend for thermal transport mediated by the three-phonon process. The weak temperature dependence in *θ*-TaN indicates the dominant role of pronounced phonon -defect scattering over higher-order phonon scattering in the experimental samples, as the latter alone would lead to a temperature dependence stronger [35] than *T*^−^^1^. As shown in Fig. 3c, we found that the presence of a mere 0.005% of nitrogen vacancies would suppress thermal conductivity by ∼40% compared to that of defect-free *θ*-TaN (Fig. S6). This strong suppression originates from on-site atomic-mass perturbations and force-constant perturbations (bonding distortions) around the vacancy sites, which strongly suppress thermal conductivity [36]. The measured temperature dependence can be interpreted through a quantitative comparison of scattering rates between these phonon scattering mechanisms (Fig. S7). The phonon -defect scattering rates of nitrogen vacancies are clearly larger than those associated with four-phonon scattering in the frequency range of ∼2.5–7.5 THz, covering phonons with a dominant contribution to the room-temperature thermal conductivity of *θ*-TaN. Our NPD refinement provides a statistical and average concentration of nitrogen vacancies of 1%, although the vacancies may not be evenly distributed throughout the as-synthesized *θ*-TaN crystals. This concentration is more than sufficient to account for the reduction of thermal conductivity to 502 W m^−^^1^ K^−^^1^ at room temperature. We noticed that at temperatures higher than 450 K, the thermal conductivity of our *θ*-TaN approaches that of BAs reported by Tian *et al*. [7] with a weaker decreasing trend, which highlights its potential application for heat dissipation in high-temperature scenarios. Below 150 K, the thermal conductivity increases with temperature since the phonon MFP is constrained by both phonon–boundary scattering from the confined sample thickness and phonon -defect scattering from nitrogen vacancies. In this regime, the thermal conductivity scales fundamentally with the temperature-dependent volumetric heat capacity (Fig. S8).

The reported presence of arsenic vacancies in BAs is analogous to the nitrogen vacancies found in *θ*-TaN. However, an equivalent concentration of arsenic vacancy in BAs induces a substantially greater reduction in thermal conductivity than the suppressive effects of nitrogen vacancies observed in *θ*-TaN [37]. We attribute the discrepancy in vacancy-induced phonon -defect scattering between *θ*-TaN and BAs to the different atomic masses of the missing species. In compounds with a large mass ratio, the heavy atoms dominate the vibrational motion of the heat-carrying acoustic modes, which contribute more to thermal conductivity [38]. Our theoretical calculations provide an illustration of the fundamental effect of vacancies on phonon thermal transport. These calculations point to a likely higher thermal conductivity for defect-free and bulk *θ*-TaN single crystals, which is promising for applications in thermal management.

In the following, we concentrated on characterizing the electrical transport of the synthesized *θ*-TaN, comparing with experimental data reported for single crystals sharing the same crystal structure, including WC [39], molybdenum phosphide (MoP) [40], and zirconium telluride (ZrTe) [41]. We performed measurements on the electrical conductivity, electronic carrier concentration, and mobility via the van der Pauw method to eliminate the effect of contact resistance. The setup is schematically illustrated in Fig. 3a, with detailed information provided in the Methods and Fig. S9. The electrical resistivity increases with temperature (Fig. 3b), showing a typical behavior for metallic materials, as the strength of phonon -electron interactions increases with temperature. The increasing trend of temperature-dependent resistivity is consistent with literature reports for WC, MoP, and ZrTe. The experimental value is lower than that of *θ*-TaN polycrystals [34,42] but remains comparable to that of bismuth [43] and higher than the value reported in titanium [44]. Moreover, this resistivity is notably lower than the in-plane electrical resistivity in graphite, and three orders of magnitude lower than graphite’s cross-plane electrical resistivity [45]. Hall effect measurements reveal that electrons are the dominant carrier for charge transport, which originates from the electron pockets along the *Γ-A* direction [15]. The carrier concentration increases slightly with rising temperature (Fig. 3c), ranging from 10^19^ cm^−^^3^ to 10^20^ cm^−^^3^. These values are nearly three orders of magnitude smaller than those of conventional metals due to relatively smaller band overlap and the tiny size of Fermi pockets [15]. Moreover, the carrier concentration of *θ*-TaN is lower than that of WC and MoP, which is consistent with the lower electronic density of states near the Fermi level and consequently supports a significantly suppressed rate of phonon -electron scattering. These combined results provide compelling experimental validation for the electronic origin of the ultrahigh thermal conductivity observed in *θ*-TaN. The measured electronic mobility (Fig. 3d) is higher than that typically observed in doped semiconductors with the same electron concentration [46], since dopant scattering would severely reduce the electron mean free path. Although the electronic carrier mobility of *θ*-TaN is lower than that of WC and ZrTe due to scattering from nitrogen vacancies, the measured values are one order of magnitude higher than those reported for MoP. This substantial difference implies a longer electronic carrier mean free path in *θ*-TaN, which is indicative of intrinsically weaker phonon -electron interactions.

**Figure 4.**
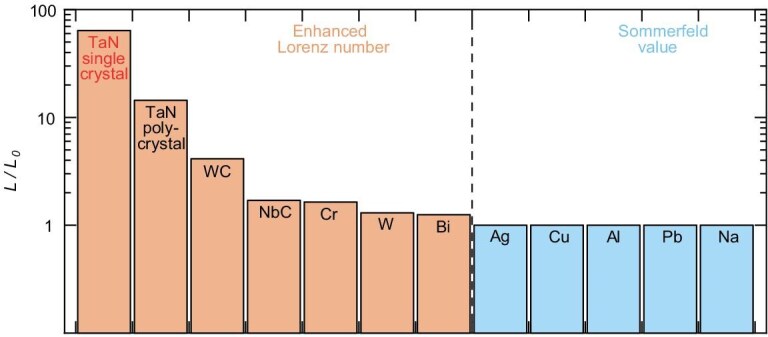
Ratio of the Lorenz number to the Sommerfeld value (*L/L_0_*) in the *θ*-TaN single crystal at room temperature, compared to reported values in *θ*-TaN polycrystal [34], WC [13], NbC [13], chromium [61], tungsten [11], bismuth [62], and other conventional metals [63–65].

Based on the thermal conductivity and electrical resistivity measured in our synthesized *θ*-TaN single crystal, we extracted a Lorenz number of 64*L_0_* at room temperature (Fig. 4), where *L_0_* stands for the Sommerfeld value (2.44 × 10^−^^8^ W Ω K^−^^2^). The calculated Lorenz number in *θ*-TaN currently represents the highest recorded value among all known metallic materials to date. The strikingly large Lorenz number exhibited a pronounced deviation from the Wiedemann–Franz law, which is applicable when electron diffusion dominates the heat transfer. This significant deviation indicates that either electrons carry exceptionally more heat than expected or alternative heat carriers dominate the thermal transport [11–13]. Specifically, lattice contribution overwhelmingly dominates thermal transport in *θ*-TaN, where the electronic contribution accounts for only 1.6% of the total thermal conductivity.

Outstanding thermal properties position *θ*-TaN as a promising heat dissipation material for electronic and industrial applications. In high-temperature environments, excellent thermal stability is a critical requirement to prevent material degradation due to oxidation [47]. We therefore conducted thermogravimetry analysis (TGA) to assess *θ*-TaN’s oxidation resistance, with the results shown in Fig. S10. The measured oxidation temperature of *θ*-TaN is 740°C, a value substantially higher than that of copper, a widely used material for heat dissipation [48]. This value also exceeds that of common carbon-based heat dissipation materials, such as graphene [49] and diamond [50], further situating *θ*-TaN as a promising candidate for high-temperature thermal management applications. Furthermore, *θ*-TaN exhibits better thermal stability compared to other cemented carbides [51], along with reported exceptional hardness [52].

We should note that the electrical transport properties of *θ*-TaN are not comparable to those of highly conductive conventional metals, such as aluminum or copper, which may constrain its utility in certain high-current applications like power transmission lines and high-current devices. Nevertheless, the combination of outstanding mechanical properties, along with exceptional thermal conductivity, makes *θ*-TaN a compelling choice for a variety of high-performance applications, such as advanced transistor technologies [53], high-power-density electronics [54], random-access memory chips [55], and aerospace engineering [56].

Achieving viable practical applications of *θ*-TaN requires the development of scalable synthesis methodologies while consistently maintaining excellent crystal quality. Although our high-pressure strategy uniquely enables the stabilization and growth of phase-pure *θ*-TaN single crystals with low vacancy concentrations, providing a roadmap for scalable production, it is limited by high operational cost, intricate preparation procedures, and inconsistent crystal dimensions. Industrially mature techniques, such as atomic layer deposition (ALD) and plasma-enhanced chemical vapor deposition (PECVD), would offer strong potential for scalable production of *θ*-TaN. Specifically, in ALD, we may achieve suitable lattice strain for stabilizing the *θ*-TaN phase by appropriate selection of substrates [57]. Furthermore, PECVD enables the tuning of plasma conditions and vapor pressures to stabilize the hexagonal *P*${\mathrm{\bar{6}}}$*m*2 structure [58]. However, achieving the elevated nitrogen chemical activity for *θ*-TaN in ALD requires exceptionally precise control of growth parameters [59], while for PECVD, plasma-induced reactions introduce additional complexity in precursor decomposition and phase-pure growth [58]. Such technical limitations restrict precise stoichiometric control and significantly complicate the attainment of phase-pure *θ*-TaN. Therefore, substantial further research is required to develop integrated synthesis strategies to effectively achieve scalable production with high crystal quality.

## SUMMARY

This investigation takes an important step in the experimental pursuit of ultrahigh thermal conductivity in metallic materials by synthesizing the *θ*-TaN single crystal and characterizing its thermal transport. The measured thermal conductivity exceeds that of all metallic thermal conductors, even in the presence of nitrogen vacancies and strong phonon -defect scattering. The ultrahigh thermal conductivity originates from the combination of a large acoustic-optical phonon energy gap and weak phonon -electron scattering. Our first-principles calculations indicate that reducing defects in *θ*-TaN could increase thermal conductivity by a factor of two, which could be achieved by fine-tuning the crystal growth process in the future. The findings significantly pave the way for a new generation of high-performance thermal management materials.

## METHODS

### X-ray and neutron diffraction experiments

The crystal structure of the final nitride product was examined by an X-ray diffractometer equipped with a Cu target. Neutron powder diffraction (NPD) measurements were also performed at the neutron beamline of the China Mianyang Research Reactor (CMRR), the wavelength of the incident neutron beam is λ = 1.5925 Å. Crystal structural refinements were conducted using either the GSAS or FULLPROF program, respectively, based on both the XRD and NPD data. In order to collect the single-crystal XRD pattern of single crystals, hundreds of plate-like crystals were mounted on a specimen holder with the same crystallographic orientations.

### Morphology characterizations

We characterized the sample’s morphology and structure using scanning electron microscopy (SEM) and high-resolution transmission electron microscopy (HRTEM). Before SEM measurement (Thermo Fisher Scientific, Apreo 2S), the sample powder was placed on a silicon wafer and fixed with carbon tape. Before the HRTEM characterization (Technai, FEI Spirit T12), the *θ*-TaN thin film was prepared using a focused ion beam (FIB) and transferred to a copper grid. Moreover, we also conducted selected-area electron diffraction (SAED) on the prepared sample, and the diffraction patterns correspond with predicted spots along the [001] zone axis.

### Electrical measurements

We performed electrical measurements on the synthesized *θ*-TaN to explore its charge transport. Before the measurement, *θ*-TaN powders were sintered into a small cylinder, with a diameter of 4.5 mm and a thickness of 1 mm, for connecting indium electrodes. We used the van der Pauw method to eliminate the effect of contact resistance. The measurements were carried out in a physical property measurement system (Quantum Design, PPMS-9), equipped with a nanovoltmeter (Keithley, 2182) and a current source (Keithley, 2400). The current was fixed at 10 μA. For Hall effect measurement, the magnetic field was swept over the −1 T to +1 T range (Fig. S9).

### Thermogravimetry (TGA) analysis

We explored the thermal stability of the synthesized *θ*-TaN using TGA (Mettler-Toledo, Switzerland), as shown in Fig. S10. Before the experiment, the sample powder was placed into an Al_2_O_3_ crucible, which was subsequently fixed on the sample holder. The measurement was conducted in air atmosphere from room temperature to 1200°C. The fully oxidized products were identified as Ta_2_O_5_ using powder XRD.

## Supplementary Material

nwag106_Supplemental_File
