# Supplementary material for: High thermal conductivity in metallic θ-TaN single crystals
Source: Natl Sci Rev. 2026 Feb 14;13(8):nwag106. doi: 10.1093/nsr/nwag106 (PMC13131223; doi:10.1093/nsr/nwag106)
Supplement: nwag106_Supplemental_File [file nwag106_supplemental_file.pdf]

Supplementary data for

High thermal conductivity in metallic *θ*-TaN single crystals

Yizhe Liu[1,†], Xuefeng Zhou[2,3,†], Guijian Pang[4,5,†], Chao Gu[2], Guozhu Song[2], Jian Chen[2], Jesús Carrete[6], Leiming Fang[7], Shanmin Wang[2,*], Wu Li[4,8,*], and Bo Sun[1,9,*]

[1]Tsinghua SIGS, Tsinghua University, Shenzhen 518055, China;
[2]Department of Physics, Guangdong Basic Research Center of Excellence for Quantum Science, and State Key Laboratory of Quantum Functional Materials, Southern University of Science and Technology, Shenzhen 518055, China;
[3]Center for High Pressure Science & Technology Advanced Research, Beijing 100094, China;
[4]Eastern Institute for Advanced Study, Eastern Institute of Technology, Ningbo 315200, China;
[5]Department of Applied Physics, The Hong Kong Polytechnic University, Hong Kong, China;
[6]Instituto de Nanociencia y Materiales de Aragón, CSIC-Universidad de Zaragoza, Zaragoza E-50009, Spain;
[7]Key Laboratory for Neutron Physics, Institute of Nuclear Physics and Chemistry, China Academy of Engineering Physics, Mianyang 621999, China;
[8]Institute for Advanced Study, Shenzhen University, Shenzhen 518060, China;
[9]Guangdong Provincial Key Laboratory of Thermal Management Engineering & Materials, Shenzhen 518055, China

[*]**Corresponding authors.** E-mails: sun.bo@sz.tsinghua.edu.cn; wu.li@eitech.edu.cn; wangsm@sustech.edu.cn
[†]Equally contributed to this work.

This file contains the following content:

Notes S1 – S3

Figures S1 – S10

Tables S1 – S4

Note. 1 Sample preparation

We exploited a favorable route to synthesize θ-TaN from a reaction between high-purity Ta powder (Ta, Thermo Fisher Scientific, 99.97%) and sodium amide ($NaNH_2$, Thermo Fisher Scientific, 99%) under high-pressure and high-temperature conditions. The starting materials were homogeneously mixed with a molar ratio of Ta:$NaNH_2$=1:1.5 and subsequently compacted into a pellet of 10.8 mm in diameter and 3 mm in height, which was encapsulated by a molybdenum capsule and then assembled with cell parts. We utilized an excessive amount of $NaNH_2$ in order to provide a nitrogen-rich reaction environment, which compensated for volatilization losses and created a nitrogen-rich atmosphere that counteracts nitrogen atom volatilization at high temperatures [1,2]. Due to the sensitivity of $NaNH_2$ to air, these procedures were carried out in a glovebox under an argon atmosphere. Before the synthesis, we heated the starting materials with the assemblies at 500 K in a nitrogen-filled tube furnace for 2 h to eliminate oxygen contamination and possible oxidation during the synthesis process. We performed high-pressure synthesis in a DS 6×10 MN cubic apparatus installed at the High-Pressure Lab of SUSTech with well-calibrated temperature and pressure [3]. During experiments, we first compressed the cell to the target pressure of 5 GPa, followed by heating the sample cell to the desired temperature of 1700 K. We reduced the crystal growth rate with a three-stage heating process as documented in our prior study [4]. This approach effectively suppresses fluctuations in elemental concentrations, which may arise from excessively rapid crystal formation [5]. The resulting stable growth environment promotes a more uniform distribution of tantalum and nitrogen within the crystal lattice. At the target temperature, the cell was soaked for 180 min for sufficient nitrogendation of Ta powder. In the following, we employed a controlled, slow cooling rate of 20 K per minute prior to quenching to minimize residual thermal stress in the material. This precise control was implemented to prevent uneven lattice contraction arising from abrupt temperature changes. Although such abrupt changes may not significantly alter the overall stoichiometry, they can induce localized lattice imperfections, such as vacancies and dislocations, and subsequently compromise the structural uniformity of the synthesized crystals [6]. To further suppress the presence of nitrogen vacancy, we conducted high-temperature annealing at 1000 K in the nitrogen atmosphere for 2 h after crystal growth, which enabled atomic diffusion to fill lattice vacancies [7]. The obtained products were purified

with diluted nitric acid to remove byproducts and unreacted starting materials.

Note. 2 Time-domain thermoreflectance (TDTR)

2.1 TDTR setup

We performed TDTR measurements to determine the thermal conductivity Λ of the synthesized θ-TaN. Briefly, we split the femtosecond laser (Coherent, Chameleon Ultra II) with a repetition rate of 80 MHz and a wavelength of 785 nm into two beams by a polarizing beam splitter. We modulated the probe beam at an audio frequency of 200 Hz by a mechanical chopper (Thorlabs, MC2000B) to improve the signal-to-noise ratio. TDTR signals with respect to the delay time between pump and probe beams were demodulated through a lock-in amplifier (SRS, SR844), which were later fitted using a three-layer thermal model to extract thermal properties. We used a longpass filter and two bandpass filters to eliminate the artifacts induced by the leaked pump beam. Temperature-dependent thermal conductivity was measured in a cryostat (Physike, S500) with a temperature controller (Cryocon, Model 22C).

Before TDTR measurements, we deposited an Al layer of 103 nm on the sample surface, serving as the thermoreflectance transducer, via magnetron sputtering (Shanghai Shilu, UHVSPUTTER-350L). The thermal conductivity of the Al transducer was calculated from the measured electrical resistivity, according to the Wiedemann-Franz law. Given the thickness of the θ-TaN is ~3 μm, we employed a high modulation frequency f = 10.1 MHz for the pump beam through an electro-optic modulator to confine heat flow within the sample. The corresponding thermal penetration depth $d = \sqrt{\Lambda/\pi C f}$ =1.74 μm, where C is the volumetric heat capacity. This specific experimental configuration ensures that our measurements exhibit maximum sensitivity to the thermal conductivity of θ-TaN along the c-axis. We used a focused beam radius of 6 μm for both pump and probe beams, considering the crystal's lateral size of ~30 μm. We used a 70 mW power of the pump beam and 5 mW power of the probe beam, ensuring the steady-state temperature rise is less than 1 K.

2.2 Oxidation layer on the sample surface

To explore the electronic structures of the synthesized θ-TaN, we performed XPS experiments (Physical Electronics, PHI 5000 VersaProbe II) with a focus on Ta4f and N1s states. The

spin−orbit splitting contributes to a doublet of Ta4f with two components of $4f_{7/2}$ and $4f_{5/2}$ (Fig. S1a). In the high-energy range, an additional Ta4f doublet is also observed, which arises from a Ta_2O_5 surface layer of the sample due to the surface oxidation effect [8,9]. Notably, the Ta4f doublet lines ($Ta4f_{7/2}$ ~ 22.8 eV) of θ-TaN show a slight shift to lower binding energies relative to the standard nitride (Ta $4f_{7/2}$ ~23 eV), suggesting the presence of minimal N vacancies [10]. This can also be demonstrated by the N1s state as θ-TaN has a greater N1s binding energy of 397.6 eV than that of the standard nitride (i.e., 397 eV) (Fig. S1b). The broad peaks around 401.6 eV and 404.7 eV can be assigned to Ta $4p_{3/2}$ states originating from θ-TaN and Ta_2O_5, respectively. To get further information on the Ta_2O_5 contaminant, we then conducted the XPS depth profiles analysis by employing surface etching treatment with Ar+ ion beam since XPS signals are inherently surface-sensitive, with most photoelectrons escaping from the sample surface within a 10 nm depth. We found that the peak intensity of the Ta-O doublets can be profoundly reduced as the sample surface was etched by Ar^+ irradiation (Fig. S1c & 1d). These doublets vanished at an etching depth of 60 nm, indicating the thickness of the surface oxidation layer and no apparent oxygen substitution in underlying θ-TaN crystals. We should note that no oxygen-related information is observed in our XRD and NPD data, since the sufficiently thin oxidation layer has a negligible influence on the patterns acquired.

We fitted our TDTR data using a multi-layered thermal model [11,12], considering that there exists a 60 nm-thick oxidation layer on the sample surface. The thermal model consisted of three layers, including a top Al transducer, oxidation layer, and θ-TaN, where the impact of the Al/oxidation and oxidation/θ-TaN interfaces was calculated in the thermal conductivity of the oxidation layer. We simultaneously fitted the thermal conductivity of the oxidation layer and θ-TaN with TDTR data acquired from 300 ps to 4 nm, since both of them showed considerable sensitivity to our measurements. The volumetric heat capacity of the oxidation layer was calculated as the average of θ-TaN and Ta_2O_5 [13], whose impact on our TDTR measurements was considered in uncertainty calculations.

2.3 Sensitivity analysis

The sensitivity S of our TDTR measurements to parameter α is defined as

$$S_a = \frac{\partial \ln(-\frac{V_{in}}{V_{out}})}{\partial \ln a},$$

where $-\frac{V_{in}}{V_{out}}$ is the TDTR signal. We used a 10.1 MHz frequency to modulate the pump beam, and the radius of the TDTR beams was focused to 6 μm. Calculated sensitivity of our TDTR measurement at 300 K is provided in Fig. S2. The measurement conditions made our analysis more sensitive to thermal conductivity along the c-axis, while still exhibiting a discernible sensitivity along the a-axis. Although θ-TaN was predicted to have anisotropic thermal conductivities with a ratio of 82.6 % [14], the anisotropy is difficult to experimentally distinguish within the measurement uncertainty. Moreover, the small sample dimensions (thickness of ~3 μm and the lateral size of ~ 30 μm) limited the modulation frequency and spot size in our TDTR measurements, which also made it challenging to measure the anisotropic thermal conductivity. Therefore, we assumed that defect-phonon scattering and boundary-phonon scattering affect thermal conductivity equally for both directions, and we employed an isotropic model to fit TDTR signals in our measurements. High sensitivity values of θ-TaN's thermal conductivity indicated reliable TDTR measurements with minimal uncertainties. The sensitivity to the thermal conductivity of the oxidation layer was also notable for TDTR signals, which served as another fitting parameter.

2.4 Uncertainty

The experimental uncertainty of our TDTR measurements to the thermal conductivity Λ of θ-TaN can be calculated using the following equation

$$\frac{\Delta\Lambda}{\Lambda} = \sqrt{\sum(\frac{S_a}{S_\Lambda}\frac{\Delta a}{a})^2 + (\frac{S_\varphi}{S_\Lambda}\Delta\varphi)^2},$$

where φ denotes the phase of TDTR signals. The calculated sensitivities to all parameters at a delay time of 3 ns is detailed in Supplementary Table S2, where h denotes the thickness, C represents the volumetric heat capacity, and w_0 is the radius of TDTR laser beams. The uncertainties we used for each parameter used in our thermal model are provided in Table S3. The calculated experimental uncertainties corresponding to the measured thermal conductivity of θ-TaN for all temperatures are systematically documented in Table S4.

2.5 Thermal conductivity of the oxidation layer

The depth profile of X-ray photoelectron spectroscopy (XPS) analysis identified that the oxidation layer had characteristic bonding energies for both TaN and Ta_2O_5. Our TDTR experiments also measured the temperature-dependent thermal conductivity of the oxidation layer. The results are displayed in Fig. S3. The thermal conductivity increases with rising temperature, showing a typical trend of amorphous materials. The room-temperature value of 4.52 W m^{-1} K^{-1} is higher than the reported value of tantalum oxide films [15], attributed to the presence of TaN.

2.6 Thermal conductivity of different *θ*-TaN crystals

Since the concentration of nitrogen vacancies would distribute unevenly in the synthesized *θ*-TaN, we performed TDTR measurements on four different samples with similar lateral sizes at room temperature. The measured thermal conductivity ranges from 442 W m^{-1} K^{-1} to 502 W m^{-1} K^{-1}, and the inconsistency is attributed to different concentrations of nitrogen vacancy. TDTR data fitting, along with optical images of the measured samples, is provided in Fig. S4.

2.7 Uniformity of the synthesized *θ*-TaN crystals

To characterize the uniformity of the synthesized *θ*-TaN crystals, we performed thermal mapping on the *θ*-TaN single crystal that exhibited the highest thermal conductivity. The synthesized *θ*-TaN single crystals have a lateral size of ~30 μm, while the beam diameter of our TDTR measurements is focused at 12 μm. Based on these dimensional constraints, we performed thermal mapping by measuring three distinct points across the selected sample. As displayed in Fig. S5, the results show that the thermal conductivity remains spatially uniform across the sample, with all measured values falling within the experimental uncertainty. This consistency provides robust evidence for the excellent structural uniformity of the synthesized crystal.

Note. 3 First-principles calculations

3.1 Methodology and computational details

The lattice thermal conductivity tensor (Λ_{ph}) can be expressed in the framework of the linearized Boltzmann transport equation (BTE) as [16]:

$$\Lambda_{\mathrm{ph}} = \frac{1}{k_B T^2 \Omega Z} \sum_{\lambda} n_{p\mathbf{q}}^0 (n_{p\mathbf{q}}^0 + 1)(\hbar\omega_{p\mathbf{q}})^2 \boldsymbol{v}_{p\mathbf{q}} \otimes \boldsymbol{F}_{p\mathbf{q}},$$

where p and $\mathbf{q}$ represent all phonon branches and phonon wave vectors, respectively, while $\omega_{p\mathbf{q}}$ and $\boldsymbol{v}_{p\mathbf{q}}$ denote the angular frequency and group velocity of the phonons indexed by p and $\mathbf{q}$, respectively. $\boldsymbol{F}_{p\mathbf{q}}$ is the mean free displacement given by [16]:

$$\boldsymbol{F}_{p\mathbf{q}} = \tau_{p\mathbf{q}}(\boldsymbol{v}_{p\mathbf{q}} + \Delta_{p\mathbf{q}}),$$

where $\tau_{p\mathbf{q}}$ is the phonon lifetime, the inverse of which is the scattering rate taking into account all of the possible phonon scattering mechanisms that determine Λ_{ph}. Here for semimetal θ-TaN, we will include three-phonon (3ph), four-phonon (4ph), phonon-isotope (ph-iso), and phonon-electron (ph-el) scattering. Additionally, due to the fact that defects like nitrogen vacancies are created with certain density during the growth processes and the finite character of real samples, we additionally consider phonon-defect (ph-def) and phonon-boundary (ph-bd) scattering in our calculations:

$$\frac{1}{\tau_{p\mathbf{q}}} = \frac{1}{\tau_{p\mathbf{q}}^{\mathrm{3ph}}} + \frac{1}{\tau_{p\mathbf{q}}^{\mathrm{4ph}}} + \frac{1}{\tau_{p\mathbf{q}}^{\mathrm{ph-is}}} + \frac{1}{\tau_{p\mathbf{q}}^{\mathrm{ph-el}}} + \frac{1}{\tau_{p\mathbf{q}}^{\mathrm{ph-de}}} + \frac{1}{\tau_{p\mathbf{q}}^{\mathrm{ph-bd}}}.$$

The detailed expressions for these types of scattering rates can be found in Refs. [17–24]. The $\Delta_{p\mathbf{q}}$ terms, which describe the departure from the relaxation-time approximation (RTA), contain a linear combination of $\boldsymbol{F}_{p\mathbf{q}}$ vectors [16]. Therefore, we can obtain the exact $\boldsymbol{F}_{p\mathbf{q}}$ for each phonon mode by the iterative approach starting from the RTA. In the current calculations, four-phonon scattering is dominated by umklapp processes, and thus their contributions can be neglected in the $\Delta_{p\mathbf{q}}$ terms.

The key inputs to solve the phonon BTE are harmonic (second-order) and anharmonic (third- and fourth-order) interatomic force constants (IFCs). We performed density functional theory

calculations as implemented in the VASP package [25,26] using the projector-augmented plane wave method [27] to extract all the IFCs, which were then used to obtain three-phonon and four-phonon scattering rates. For the phonon-electron scattering rate calculations, the EPW software [28] is used together with the Quantum Espresso package [29] to perform Wannier function interpolation for phonon-electron coupling matrix elements. Further computational details can be found in previous works [14,30].

The phonon-defect scattering rate calculations for nitrogen vacancy defects were executed using a modified version of the almaBTE code [31]. We first employed a 5×5×5 supercell with one N atom removed from its pristine site to perform structural relaxation as implemented in the VASP package. Phonopy package [32] was then used to extract harmonic IFCs of this defective supercell. The harmonic IFCs matrices of perfect and defective supercells were used to quantify the bonding perturbation caused by the nitrogen vacancies. We calculated the Green's functions of the perfect system on a 19×19×19 grid by employing the tetrahedron method to integrate over the Brillouin zone and then obtained the phonon-defect scattering rates on a 16×16×16 $\mathbf{q}$-grid. To reduce computation cost, a linear interpolation method was used to interpolate the entire ph-defect scattering rates to a denser 32 ×32 ×32 $\mathbf{q}$-grid. To account for phonon-boundary scattering, we employ the expression $\frac{1}{\tau_{p\mathbf{q}}^{\mathrm{ph-bd}}} = \frac{|v_{p\mathbf{q}}|}{L_{\mathrm{b}}}$ with an effective boundary mean free path L_{b}, which is equal to 7 μm in this work. This choice is larger than the 3 μm thickness of the samples. The difference can be accounted for by the fact that the above expression simply assumes a completely diffusive behavior of the boundary, which does not hold perfectly in realistic conditions, as a fraction of the incident phonons are scattered specularly. The fraction of phonons scattered specularly is often referred to as a specularity parameter *p*, which relates the effective boudary size L_{b} to actual thickness L_{s} as $L_{\mathrm{b}} = L_{\mathrm{s}}(1+p)/(1-p)$ [33]. Therefore, the assumption of completely diffusive scattering (*p*=0) represents the maximal boundary effect, known as the Casimir limit. In the current case, the effective size that can lead to good agreement with the measured thermal conductivity is 7 μm, larger than the actual sample thickness of 3 μm. This indicates that p is about 0.4. Finally, we modified the FourPhonon code [34] to additionally include the phonon-electron, phonon-defect scattering rates for the Λ_{ph} calculations.

3.2 Nitrogen-vacancy-concentration dependence of thermal conductivity at 300K

To quantify the effect of phonon-defect scattering by nitrogen vacancies on the room-temperature thermal conductivity in θ-TaN crystals, we calculated the room-temperature thermal conductivity along the a and c axes with respect to atomic percent concentrations (ranging from about 10^{-6} % to 10^{0} %). The calculated results are displayed in Fig. S5. We found that the thermal conductivity starts to rapidly decrease at concentrations above 10^{-3} %. In particular, a 0.005 % of nitrogen vacancies would suppress the thermal conductivity by ~40 % compared to that of defect-free θ-TaN.

The calculated nitrogen vacancy concentration of 0.005 % exhibits a significant discrepancy from the ensemble-averaged value of ~1 % determined by the NPD refinement. This difference is primarily attributed to the distinct sample populations analyzed by each respective method. Specifically, the NPD measurement provides an ensemble-averaged, statistical concentration, where the vacancy concentration is expected to exhibit large variation among individual θ-TaN crystals. The large number of smaller crystals and powders likely possesses a greater concentration of vacancies, and this higher defect concentration is averaged within our NPD measurements. In contrast, our TDTR measurements were conducted exclusively on select θ-TaN single crystals that possessed sufficiently large dimensions. This sample selection not only satisfied the necessary geometrical criteria for the measurement technique but also emphasized the ultrahigh thermal conductivity, which is characteristic of high-quality θ-TaN crystals. Considering the difficulty in precise defect control and quantification in materials with high thermal conductivity, the measured vacancy concentration of ~1 % is more than sufficient to account for the reduction of the thermal conductivity to 502 W m^{-1} K^{-1} at room temperature.

3.3 Scattering rate comparison between different phonon scattering mechanisms

Since both extrinsic phonon-defect scattering by nitrogen vacancies and phonon-boundary scattering exist in defect-laden θ-TaN crystals, we calculated and compared their scattering rates, together with intrinsic phonon scattering rates, including three-phonon, four-phonon, phonon-isotope, and phonon-electron scatterings, to measure their significance on thermal conductivity reduction in θ-TaN crystals. The calculated results are shown in Fig. S6. We

particularly noticed that phonon-defect scattering rates are obviously larger than those of four-phonon scattering in the frequency range from 2.5 to 7.5 THz, where the biggest phonon contribution to the thermal conductivity at 300 K comes from. On the other hand, we also found that phonon-boundary scattering is stronger than phonon-defect scattering in the frequency range below 2.5 THz, comprising phonons that dominate the thermal conductivity well below room temperature.

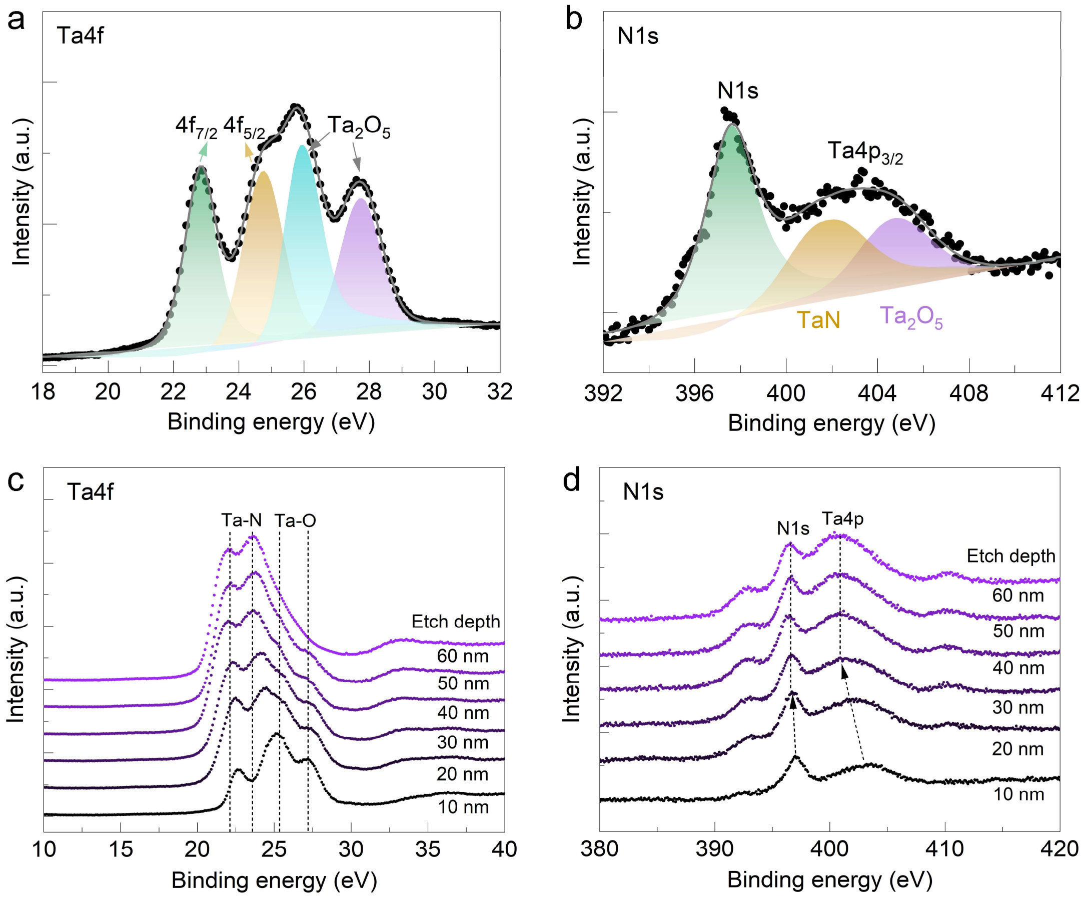

Figure S1. XPS analysis of the synthesized θ-TaN. (a) XPS spectra of Ta4f without etching. (b) XPS spectra of N1s without etching. (c) XPS depth profile of Ta4f. (d) XPS depth profile of N1s.

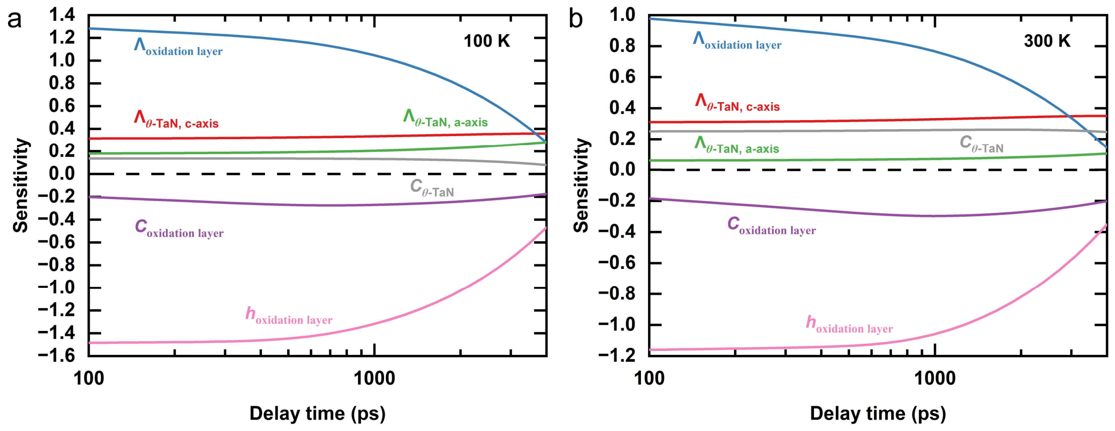

Figure S2. Sensitivity analysis of our TDTR measurement at (a) 100 K and (b) 300 K. Solid lines represent calculated sensitivity to thermal conductivity of θ-TaN along the c-axis (red), thermal conductivity of θ-TaN along the a-axis (green), thermal conductivity of the oxidation layer (blue), volumetric heat capacity of θ-TaN (gray), volumetric heat capacity of the oxidation layer (purple), and thickness of the oxidation layer (pink).

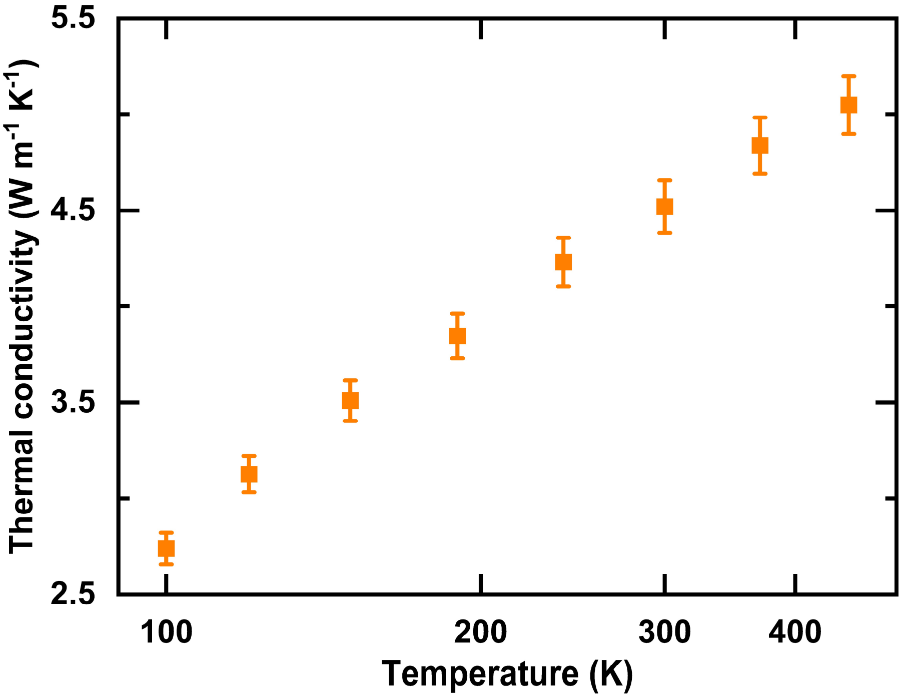

Figure S3. Temperature-dependent thermal conductivity of the oxidation layer.

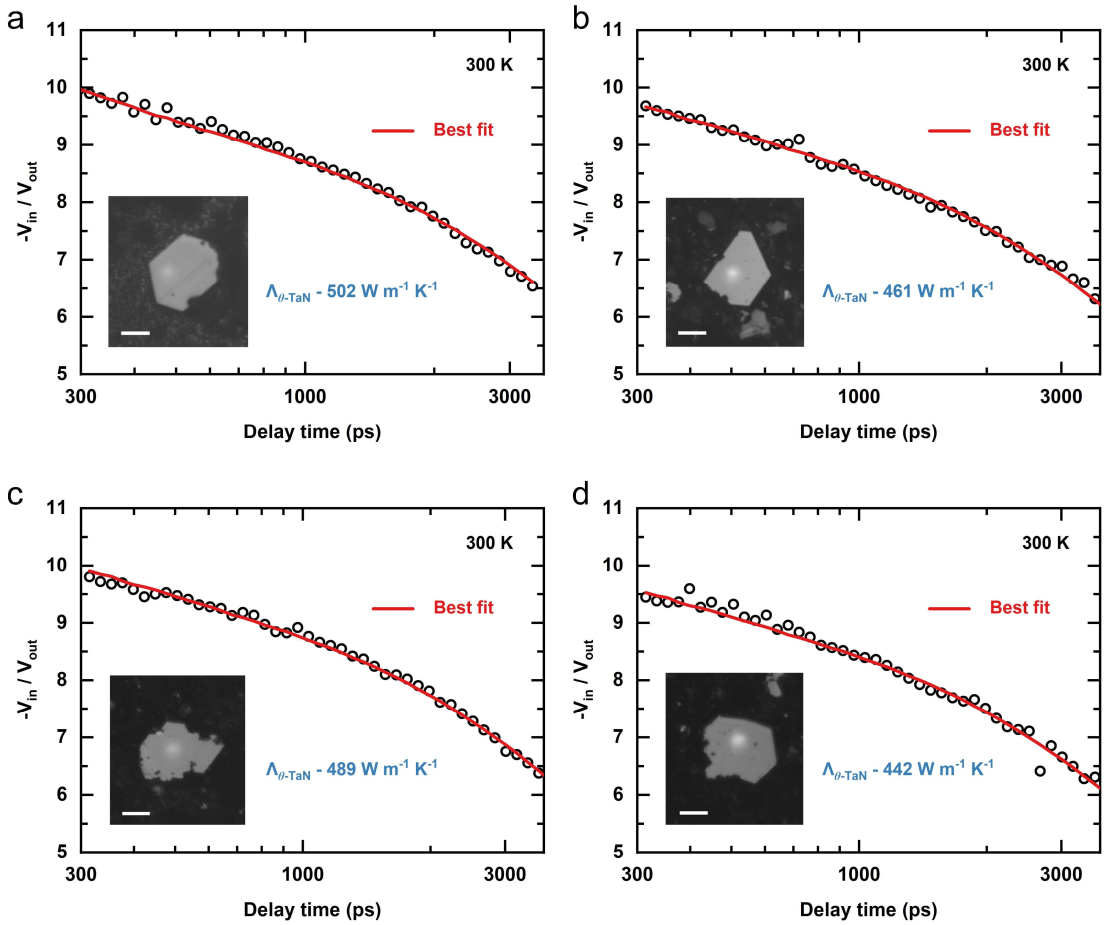

Figure S4. TDTR measurements on different θ-TaN crystals. Open black symbols show the acquired TDTR data, and red lines indicate the best fit. The insets are optical images of the measured θ-TaN crystals, where the scale bar denotes 10 μm.

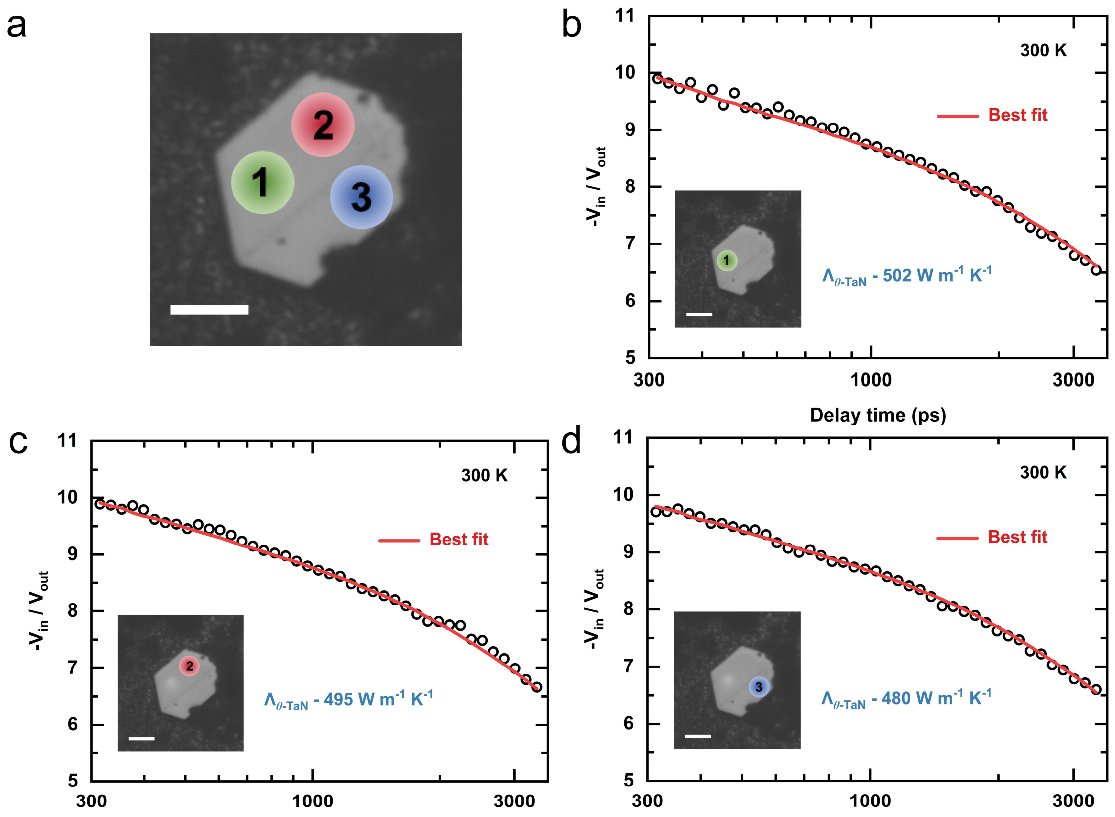

Figure S5. TDTR measurements for mapping the uniformity of the synthesized θ-TaN. Open black symbols show the acquired TDTR data, and red lines indicate the best fit. The insets are images to illustrate the measured point, where the scale bar denotes 10 μm.

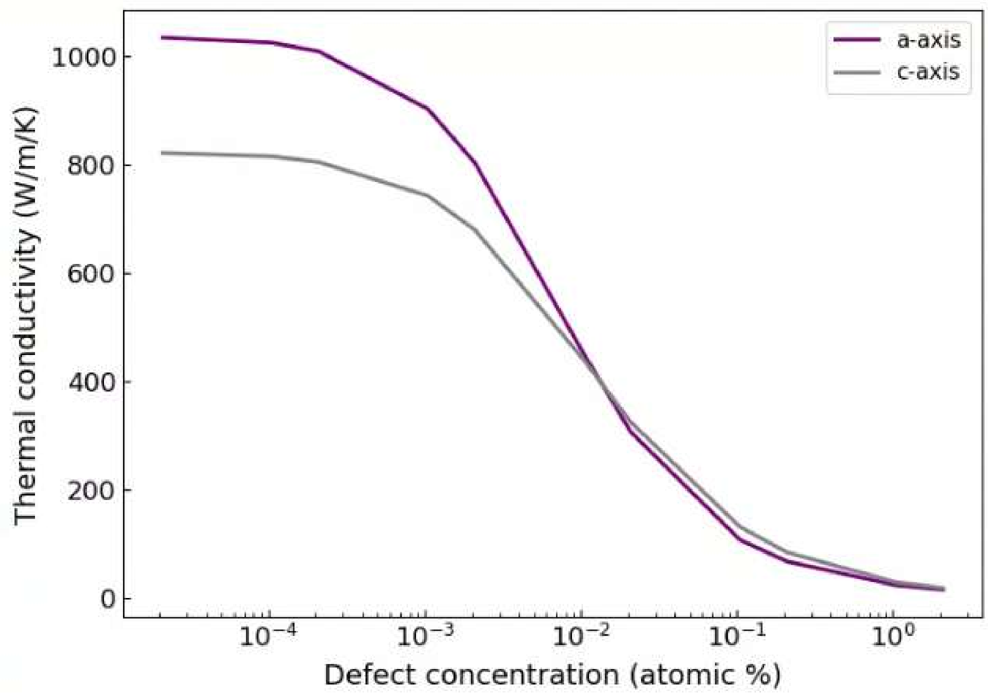

Figure S6. Calculated thermal conductivity of θ-TaN along the a-axis (purple line) and c-axis (gray line) as a function of nitrogen vacancy concentrations.

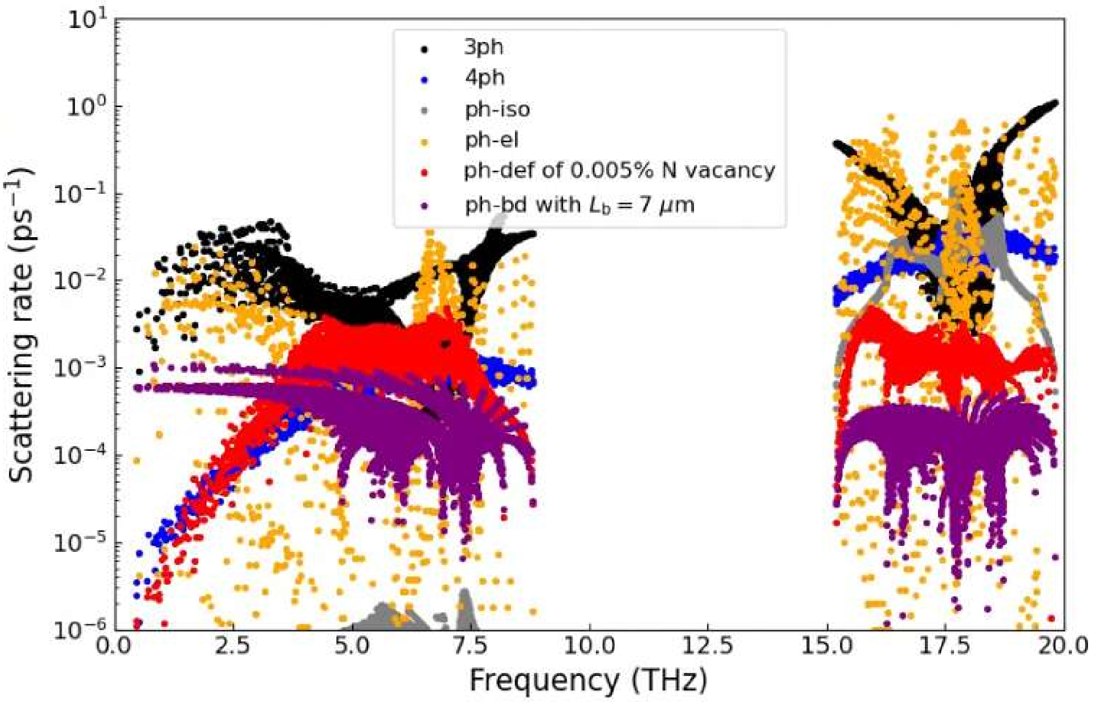

Figure S7. Calculated scattering rates for different kinds of scattering processes in θ-TaN, including three-phonon (3ph), four-phonon (4ph), phonon-isotopic (ph-iso), phonon-electron (ph-el), phonon-defect (ph-def) with 0.005% nitrogen vacancies, and phonon-boundary (ph-bd) processes with length $L_b = 7\ \mu m$.

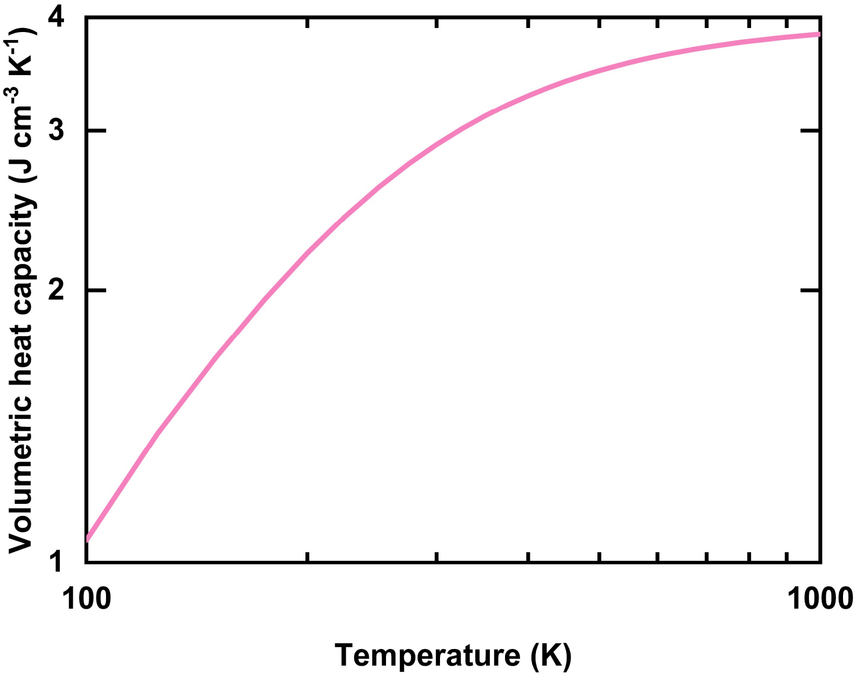

Figure S8. Volumetric heat capacity of θ-TaN from our first-principles calculations.

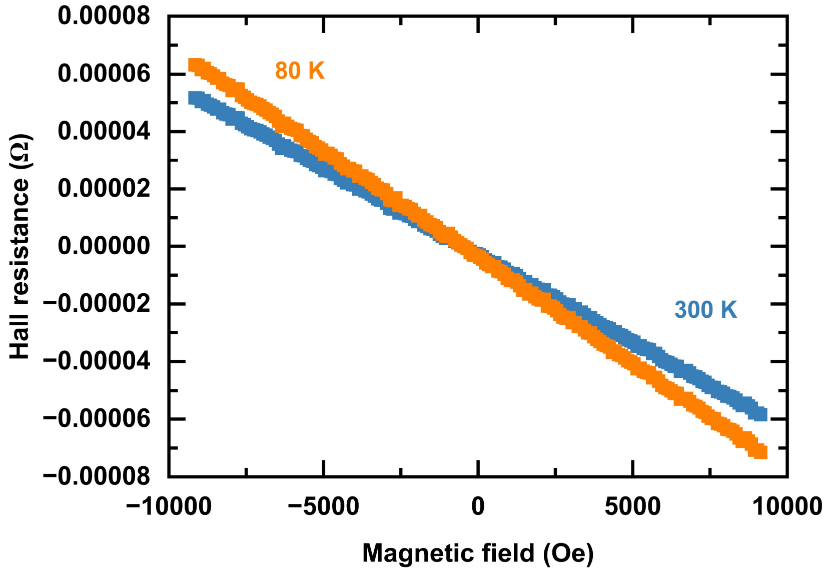

Figure S9. Hall effect measurement of the θ-TaN at 80 K (orange) and 300 K (blue). The magnetic field was swept from -1 T to +1 T.

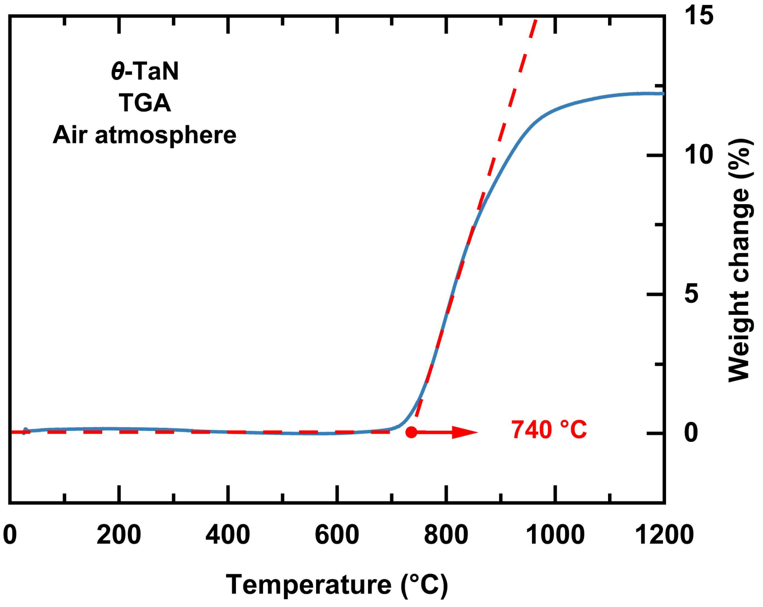

Figure S10. Thermogravimetry analysis (TGA) on the synthesized θ-TaN in the air atmosphere.

Table S1. Summary of refined crystal structure for θ-TaN by analysis of both the XRD and NPD data taken at ambient conditions.

	**θ-TaN**
Formula	$TaN_{0.99}$
Space group	hexagonal, $P\bar{6}m2$ (No. 187)
Pearson symbol	hP2
Cell parameter a, c (Å)	2.9337, 2.8832
Cell volume (Å^3)	21.491
Density (g/cm^3)	15.053
Wyckoff site	Ta: 1a (0, 0, 0), N: 1f (2/3, 1/3, 1/2)
Occupancy	Ta: 1, N: 0.99
$D_{\text{Ta-N}}$ (Å)	2.224 (2)
Uiso (Å^2)	Ta: 0.0005, N: 0.0076
VEC (electrons/cell)	9.95
wRp (%), χ^2	9.56, 3.30

Table S2. Sensitivity to thermal parameters in our TDTR measurements at 3 ns

	$S_{h_{Al}}$	$S_{\Lambda_{Al}}$	$S_{C_{Al}}$	$S_{h_{oxidation}}$	$S_{\Lambda_{oxidation}}$	$S_{C_{oxidation}}$	S_{w_0}	S_{φ}	$S_{C_{\theta-TaN}}$	$S_{\Lambda_{\theta-TaN}}$
**450 K**	-0.705	+0.038	-0.752	-0.488	+0.237	-0.234	-0.101	10.01	+0.292	+0.416
**370 K**	-0.753	+0.039	-0.799	-0.549	+0.293	-0.239	-0.117	10.21	+0.274	+0.429
**300 K**	-0.795	+0.039	-0.841	-0.555	+0.316	-0.234	-0.133	10.69	+0.255	+0.442
**240 K**	-0.851	+0.038	-0.896	-0.628	+0.381	-0.229	-0.162	11.19	+0.226	+0.511
**190 K**	-0.963	+0.039	-1.005	-0.746	+0.502	-0.226	-0.199	11.45	+0.181	+0.521
**150 K**	-1.067	+0.038	-1.106	-0.852	+0.606	-0.220	-0.245	11.69	+0.131	+0.543
**120 K**	-1.049	+0.036	-1.088	-0.781	+0.547	-0.212	-0.278	11.52	+0.111	+0.593
**100 K**	-1.043	+0.038	-1.084	-0.751	+0.522	-0.205	-0.297	11.46	+0.099	+0.617

Table S3. Uncertainty for thermal parameters in our thermal model

	$h_{oxidation}$	$C_{oxidation}$	h_{Al}	Λ_{Al}	C_{Al}	$C_{\theta-TaN}$	w_0	$\Delta\varphi$
**Uncertainty**	±5 %	±5 %	±1 %	±10 %	±2 %	±2 %	±5 %	0.001

Table S4. Calculated uncertainty for the measured thermal conductivity of θ-TaN

	**450 K**	**370 K**	**300 K**	**240 K**	**190 K**	**150 K**	**120 K**	**100 K**
**Uncertainty**	±7.5 %	±8.1 %	±8.0 %	±7.7 %	±8.7 %	±9.5 %	±8.2 %	±7.7 %

REFERENCES

1. Klimashin FF, Lobmaier L, Koutná N *et al.* The MoN–TaN system: Role of vacancies in phase stability and mechanical properties. *Mater Des* 2021; **202**: 109568.

2. Greenaway AL, Melamed CL, Tellekamp MB *et al.* Ternary nitride materials: Fundamentals and emerging device applications. *Annu Rev Mater Res* 2021; **51**: 591–618.

3. Zhou X, Ma D, Wang L *et al.* Large-volume cubic press produces high temperatures above 4000 Kelvin for study of the refractory materials at pressures. *Rev Sci Instrum* 2020; **91**: 015118.

4. Liu Y, Li Q, Qian Y *et al.* Thermal conductivity of high-temperature high-pressure synthesized θ-TaN. *Appl Phys Lett* 2023; **122**: 222201.

5. Liebermann RC. Multi-anvil, high pressure apparatus: a half-century of development and progress. *High Pressure Res* 2011; **31**: 493–532.

6. Berry T, Ng N, McQueen TM. Tools and tricks for single crystal growth. *Chem Mater* 2024; **36**: 4929–44.

7. Popović M, Novaković MM, Bibić NM. Annealing effects on the properties of TiN thin films. *Process Appl Ceram* 2015; **9**: 67–71.

8. Liu X, Ma GJ, Sun G *et al.* Effect of deposition and annealing temperature on mechanical properties of TaN film. *Appl Surf Sci* 2011; **258**: 1033–7.

9. Zaman A, Meletis EI. Microstructure and mechanical properties of TaN thin films prepared by reactive magnetron sputtering. *Coatings* 2017; **7**: 209.

10. Dai W, Shi Y. Effect of bias voltage on microstructure and properties of tantalum nitride coatings deposited by RF magnetron sputtering. *Coatings* 2021; **11**: 911.

11. Cahill DG. Analysis of heat flow in layered structures for time-domain thermoreflectance. *Rev Sci Instrum* 2004; **75**: 5119–22.

12. Albert Feldman. Algorithm for solutions of the thermal diffusion equation in a stratified medium with a modulated heating source. *High Temp - High Press* 1996; **31**: 293–8.

13. Friedrich A, Morgenroth W, Bayarjargal L *et al.* In situ study of the high pressure high-temperature stability field of TaN and of the compressibilities of θ-TaN and TaON. *High Press Res* 2013; **33**: 633–41.

14. Kundu A, Yang X, Ma J *et al.* Ultrahigh thermal conductivity of θ-phase tantalum nitride.

Phys Rev Lett 2021; **126**: 115901.

15. Landon CD, Wilke RHT, Brumbach MT *et al.* Thermal transport in tantalum oxide films for memristive applications. *Appl Phys Lett* 2015; **107**: 023108.

16. Li W, Carrete J, A. Katcho N *et al.* ShengBTE: A solver of the Boltzmann transport equation for phonons. *Comput Phys Commun* 2014; **185**: 1747–58.

17. Feng T, Lindsay L, Ruan X. Four-phonon scattering significantly reduces intrinsic thermal conductivity of solids. *Phys Rev B* 2017; **96**: 161201.

18. Ward A, Broido DA, Stewart DA *et al.* Ab initio theory of the lattice thermal conductivity in diamond. *Phys Rev B* 2009; **80**: 125203.

19. Liao B, Qiu B, Zhou J *et al.* Significant reduction of lattice thermal conductivity by the electron-phonon interaction in silicon with high carrier concentrations: A first-principles study. *Phys Rev Lett* 2015; **114**: 115901.

20. Chen Y, Ma J, Li W. Understanding the thermal conductivity and Lorenz number in tungsten from first principles. *Phys Rev B* 2019; **99**: 020305.

21. Feng T, Ruan X. Quantum mechanical prediction of four-phonon scattering rates and reduced thermal conductivity of solids. *Phys Rev B* 2016; **93**: 045202.

22. Katre A, Carrete J, Dongre B *et al.* Exceptionally strong phonon scattering by B substitution in cubic SiC. *Phys Rev Lett* 2017; **119**: 075902.

23. Guo G, Yang X, Carrete J *et al.* Revisiting the thermal conductivity of Si, Ge and diamond from first principles: roles of atomic mass and interatomic potential. *J Phys: Condens Matter* 2021; **33**: 285702.

24. Pang G, Meng F, Chen Y *et al.* Thermal conductivity reduction in highly-doped cubic SiC by phonon-defect and phonon-electron scattering. *Mater Today Phys* 2024; **41**: 101346.

25. Kresse G, Furthmüller J. Efficient iterative schemes for *ab initio* total-energy calculations using a plane-wave basis set. *Phys Rev B* 1996; **54**: 11169–86.

26. Kresse G, Joubert D. From ultrasoft pseudopotentials to the projector augmented-wave method. *Phys Rev B* 1999; **59**: 1758–75.

27. Blöchl PE. Projector augmented-wave method. *Phys Rev B* 1994; **50**: 17953–79.

28. Poncé S, Margine ER, Verdi C *et al.* EPW: Electron–phonon coupling, transport and superconducting properties using maximally localized Wannier functions. *Comput Phys*

Commun 2016; **209**: 116–33.

29. Giannozzi P, Baroni S, Bonini N *et al.* QUANTUM ESPRESSO: a modular and open-source software project for quantum simulations of materials. *J Phys: Condens Matter* 2009; **21**: 395502.

30. Kundu A, Chen Y, Yang X *et al.* Electron-induced nonmonotonic pressure dependence of the lattice thermal conductivity of θ-TaN. *Phys Rev Lett* 2024; **132**: 116301.

31. Carrete J, Vermeersch B, Katre A *et al.* almaBTE : A solver of the space–time dependent Boltzmann transport equation for phonons in structured materials. *Comput Phys Commun* 2017; **220**: 351–62.

32. Togo A, Tanaka I. First principles phonon calculations in materials science. *Scr Mater* 2015; **108**: 1–5.

33. Li W, Carrete J, Mingo N. Thermal conductivity and phonon linewidths of monolayer MoS_2 from first principles. *Appl Phys Lett* 2013; **103**: 253103.

34. Han Z, Yang X, Li W *et al.* FourPhonon: An extension module to ShengBTE for computing four-phonon scattering rates and thermal conductivity. *Comput Phys Commun* 2022; **270**: 108179.
